# Air in the kidney: between emphysematous pyelitis and pyelonephritis

**DOI:** 10.2349/biij.4.4.e24

**Published:** 2008-10-01

**Authors:** CH Kua, YF Abdul Aziz

**Affiliations:** 1 Department of Biomedical Imaging, Faculty of Medicine, University of Malaya, Kuala Lumpur

**Keywords:** Emphysematous pyelitis, emphysematous pyelonephritis, CT scan

## Abstract

Presence of air in the kidney can be problematic as the location of the air in different parts of the kidney greatly affects the subsequent management and outcome of the patient. We present here a case of a patient who had emphysematous pyelitis, in which CT scan was able to display presence of air only in the collecting system, thus differentiating this condition from the more fulminant emphysematous pyelonephritis. This leads to a more favourable prognosis and outcome to the patient.

## INTRODUCTION

Emphysematous (gas-forming) infections in the abdomen and pelvis represent potentially life-threatening conditions that require aggressive medical and, often, surgical management. They frequently progress rapidly to sepsis in the absence of any early therapeutic interventions [1]. However, among the entities of gas-forming infections, there is a condition called emphysematous pyelitis where the prognosis is excellent, with rapid complete recovery after medical treatment [2].

Emphysematous pyelitis is the term used to describe the presence of gas limited to the renal excretory system [1]. It is a benign condition with a low overall mortality rate as compared to emphysematous pyelonephritis [2]. Thus, it is important to distinguish between these two types of gas-forming renal infections because of the prognostic differences and, hence, the differences in clinical management.

We present a case of emphysematous pyelitis diagnosed on CT scan. Described are the imaging findings in emphysematous pyelitis and how they differ from findings in emphysematous pyelonephritis. We will further discuss the clinical management of the two entities.

## CASE REPORT

Mr SM is a 63-year-old Indian man with a history of diabetes mellitus and hypertension. He presented to the trauma and emergency department with a two-day history of dysuria, haematuria and suprapubic pain. He also reported having intermittent fever over the past week. Clinically, he was afebrile and his vital signs were stable. His abdomen was soft, revealing tenderness only at the suprapubic region.

**Figure 1:**
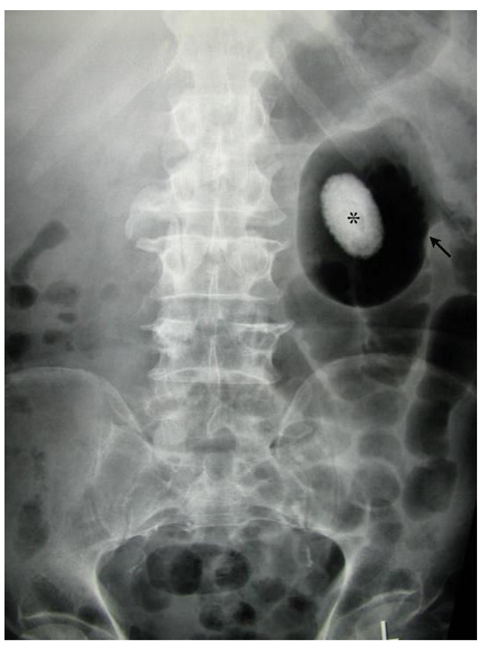
Plain abdominal radiograph showing oval radiopacity (*) surrounded by a radiolucent rim (arrow) at the region of the left kidney.

His abdominal radiograph taken in supine position revealed an oval radiopacity measuring 4 x 4cm surrounded by a radiolucent rim at the region of the left kidney (Figure 1). A provisional diagnosis of a gas-forming infection of the left kidney was made. Subsequent ultrasound revealed multiple echogenic lines associated with dirty shadowing (containing low-level echoes and reverberations) at the region of the sinus of the left kidney in keeping with gas formation. There was another echogenic focus with ‘clean’ shadow noted posteriorly in keeping with calculus. However, the exact position of the gas and calculus in relation to the left kidney and the pelvicalyceal system could not be delineated due to the presence of their shadows. At this time, it was imperative to rule out emphysematous pyelonephritis in this patient.

**Figure 2:**
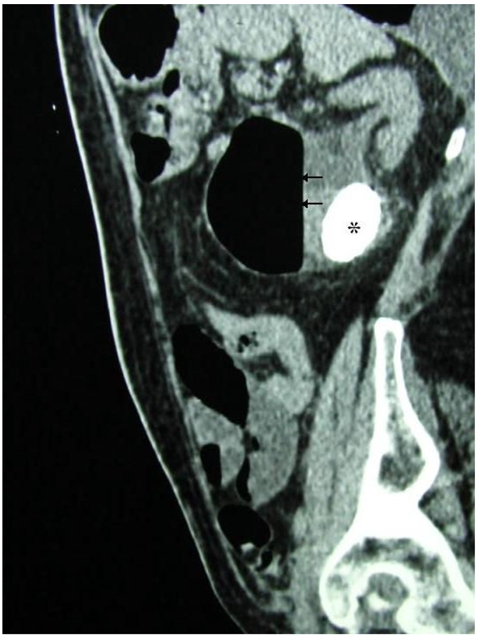
Reformatted sagittal image of contrasted CT scan of the abdomen showing a large obstructing calculus (*) at the left pelvi-ureteric junction, causing gross left hydronephrosis with gas (arrow) noted within the left pelvicalyceal system.

**Figure 3:**
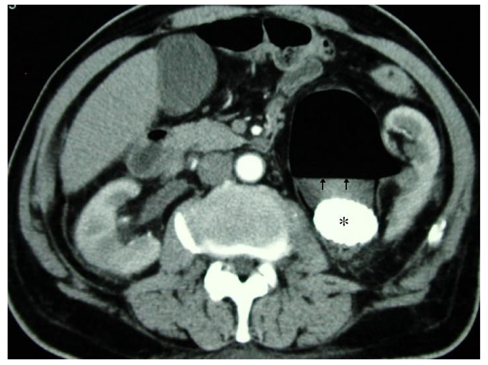
Axial image of contrasted CT scan of the abdomen showing a large obstructing calculus (*) at the left pelvi-ureteric junction, causing gross left hydronephrosis with gas (arrow) noted within the left pelvicalyceal system.

An urgent plain and contrasted CT abdomen and pelvis was performed. There was a large obstructing calculus noted at the left pelvi-ureteric junction causing gross left hydronephrosis with gas noted within the left pelvicalyceal system (Figure 2 and Figure 3). However, there were no gas pockets or fluid collections seen within the left renal parenchyma or in the left perinephric tissues. The urinary bladder showed no presence of air as well.

Diagnosis of emphysematous pyelitis was made based on the CT findings. Subsequently an urgent left nephrostomy was arranged and was done later on the day of admission. Intravenous antibiotics (IV Cefobid 2g BD and IV Tazosin 4.5g) were commenced on the same day. Patient responded favourably to the treatment with no fever recorded in the ward. A left JJ stent was inserted and the patient was subsequently discharged. ESWL (extracorporeal shock wave lithotripsy) was planned for the patient on a later date.

## DISCUSSION

Emphysematous pyelitis is a rare infection of the urinary collecting system due to gas forming bacteria [1, 3]. Emphysematous pyelitis carries a mortality rate of up to 20%, which is significantly lower than that of emphysematous pyelonephritis, which carries a mortality rate of approximately 50% [1]. It is often associated with underlying diabetes although the relationship with diabetes mellitus is lower than in emphysematous pyelonephritis [2]. Underlying poorly-controlled diabetes mellitus is present in up to 90% of patients who develop emphysematous pyelonephritis [1] compared to only 50% of patients with emphysematous pyelitis who have diabetes mellitus [4]. Emphysematous pyelitis usually accompanies urinary tract obstruction of which the cause of obstruction is usually calculi [3]. The patient in this case had both diabetes mellitus and obstructing pelvi-ureteric calculus.

The clinical manifestation of emphysematous pyelitis tends to be non-specific, similar to the clinical presentation of uncomplicated acute pyelonephritis [2]. Roy et al reported five patients, who were eventually diagnosed with emphysematous pyelitis, all had a one-week history of fever and chills at presentation. These symptoms were associated with upper-quadrant tenderness on either side depending on where the pathology was. Four of the patients had dysuria, and one had pyuria. Macroscopic haematuria was found in one patient [2]. The patient in our case presented with symptoms of urinary tract infection with fever, dysuria, haematuria and suprapubic pain; very similar to the cases documented by Roy et al except that there was no loin or upper-quadrant tenderness. On the contrary, most patients with emphysematous pyelonephritis are severely ill with chills, fever, flank pain, frequent lethargy and confusion, and multiple associated medical problems such as uncontrolled hyperglycaemia, acidosis, dehydration and electrolyte imbalance [4].

A typical feature seen in conventional radiography of emphysematous pyelitis is the presence of gas outlining the ureters and pelvicalyceal system. The rate of diagnosis with plain radiography is low because of intestinal gases [3]. In this case, due to the obstructing ureteric calculus, the presence of gas was concentrated in the dilated pelvis of the left kidney. Ultrasound findings include high-amplitude shadowing along the non-dependent surfaces that was present in this case causing obscuration of the posterior structures. The posterior shadowing is typically ‘dirty’ as opposed to the ‘clean’ shadowing caused by calculus. Kiris et al reported ultrasound findings that were different from the typical appearances. The gas appeared as a hypoechogenic area surrounded by an echogenic (calcific) rim and more echogenic central stone, thus giving the appearance of a target sign [3].

Finally, a CT scan was performed as this best delineates gas within the collecting system and helps reliably identify ureteric stones as demonstrated in this case. Based on the CT appearance, the diagnosis of emphysematous pyelonephritis could be excluded because there were no gas pockets or fluid collections within the renal parenchyma or perinephric tissue.

Gas appearance inside the urinary system can be caused by infections due to gas-producing bacteria, fistulas related to the gastrointestinal system, gas reflux from the urinary bladder, trauma and the urinary system’s interventional procedures [3]. Common bacterial causes of emphysematous pyelitis are *E. Coli*, *K. pneumonia* and *Aerobacter* [3]. No organism was cultured from the urine sample in this case although the urine microscopy revealed packed field of leucocytes and 2+ of bacteria.

Emphysematous pyelonephritis has been defined as an acute, severe, necrotising infection of the renal parenchyma and perirenal tissue, which results in the presence of gas within the renal parenchyma, collecting system, or perinephric tissue [5]. A CT classification scheme proposed by Wan et al [5] divides emphysematous pyelonephritis into two types with different prognostic significance. Type I emphysematous pyelonephritis is characterised by parenchymal destruction with streaky or mottled gas collections but no fluid collections. Type II emphysematous pyelonephritis is characterised by bubbly or loculated gas within the parenchyma or collecting system with associated renal or perirenal fluid collections that are thought to represent a favorable immune response. Based on these descriptions by Wan et al, emphysematous pyelitis falls under Type II emphysematous pyelonephritis which carries a mortality rate of 18% versus 69% for Type I [5]. In the classical Type I emphysematous pyelonephritis, a reduced immune response limits the formation of pus collection and this leads to the spread of the inflammation culminating in a fulminant course of the disease. In contrast, a better immune response in Type II emphysematous pyelonephritis causes formation of pus in the kidney leading to a slower course of the disease and a better prognosis [5, 6].

The clinical management of emphysematous pyelitis and emphysematous pyelonephritis are different. Intraparenchymal gas usually requires drainage or nephrectomy and is associated with a substantial mortality rate. In the case of emphysematous pyelitis, if gas is localised to the collecting system and no obstruction is present, antibiotic therapy alone appears to be sufficient [2].

In conclusion, we presented herewith a patient with emphysematous pyelitis. CT proved to be a good imaging modality for depicting this disease process as it is sensitive in precisely localising air within the pelvicalyceal system and eliminating air within the renal parenchyma and/or perinephric space [2], thus reliably excluding the more fulminant emphysematous pyelonephritis as a diagnosis.
